# Editorial: Metals and neurodegeneration: restoring the balance

**DOI:** 10.3389/fnagi.2015.00127

**Published:** 2015-07-02

**Authors:** Anthony R. White, Katja M. Kanninen, Peter J. Crouch

**Affiliations:** ^1^Department of Pathology, University of MelbourneParkville VIC, Australia; ^2^Neurobiology, A.I. Virtanen Institute for Molecular Sciences, University of Eastern FinlandKuopio, Finland

**Keywords:** metals, neurodegenerative diseases, Alzheimer's disease, Parkinson's disease (PD), copper, zinc, iron, iron chelating agents

There is considerable evidence that abnormal biometal homeostasis is a key feature of many neurodegenerative diseases and may have an important role in the onset and progression of disorders such as Alzheimer's (AD), Parkinson's (PD), prion, and motor neuron diseases. The role of biometals in a growing list of brain disorders is supported by evidence from a wide range of sources including molecular genetics, biochemical studies and biometal imaging. These studies have spurred a growing interest in understanding the role of biometals in brain function and disease as well as the development of therapeutic approaches that may be able to restore the altered biometal chemistry of the brain. In this Research Topic, Metals and Neurodegeneration: Restoring the Balance, we probe the biochemical basis of metal-mediated neurodegeneration, examine genetic links between metal dyshomeostasis and brain disorders, investigate metal trafficking and metal-synaptic interactions, and their role in neurodegeneration, and examine some of the key new approaches to understanding how metals drive neurodegenerative changes. We hope that these exciting insights will provide a strong platform to develop advances in therapeutics that will allow us to “restore the balance” in metal homeostasis in the brain.

One of the most prominent features across many neurodegenerative disorders is loss of metal homeostasis, and in many cases, the metal revealing the most substantial change is iron (Fe). Opening this Research Topic, Hare et al. (2013) provides an excellent overview of Fe transport and Fe-regulatory processes in the brain, and demonstrates clearly the complexity in these processes. The review describes how abnormalities in this complex process can lead to loss of Fe, which is associated with changes in neurotransmission, energy production and myelination, and is associated with diseases such as AD. Conversely, abnormal Fe handling can also lead to Fe accumulation, which is associated with AD and PD and is a major target of therapeutic developments based on Fe chelation. Expanding on Fe in neurodegeneration is the review by Muhoberac and Vidal (2013), who explore the genetic basis of Fe dyshomeostasis in hereditary ferritinopathy. The article provides a timely insight into the effects of abnormal Fe metabolism through loss of ferritin function, a key Fe-regulatory protein and how these changes can lead to ferritin accumulation, reactive oxygen species formation and oxidative stress. Mariani et al. (2013), further explores hereditary links between Fe and neurodegeneration. They describe links between Fe-specific gene variations (e.g., transferrin, hemochromatosis) and Fe regulatory proteins (ceruloplasmin and apolipoprotein E) in AD, PD and mild cognitive impairment. Despite a small cohort and rare alleles the studies provide important insights on altered Fe in neurodegeneration and illustrate that the role of metals in neurodegeneration must be examined in association with genetics, and disease sub-populations to gain a clear insight into the contributory role of metals in these diseases. When we think of metals and neurodegeneration, we often focus on the brain, but metal changes in the eye are also prominent in these disorders. Song and Dunaief (2013) describe the role of Fe in retinal degeneration in hereditary Fe overload disorders and the potential impact of Fe accumulation in acute macular degeneration.

Other metal changes also feature strongly in neurodegenerative diseases. Telianidis et al. (2013) provides an excellent overview of the key copper (Cu) transport proteins, ATP7a and ATP7b in cellular Cu homeostasis. They provide important insights into the cell fate when abnormal Cu trafficking occurs, as is evidenced by the genetic diseases, Menkes and Wilson disease, and more broadly in AD and prion diseases. Dringen et al. (2013) extends this to describe the essential role of Cu uptake, transport, metabolism, and export by astrocytes in the brain and how this has a major impact in neuronal survival and function. Cu is also an essential metal in synaptic function as illustrated by the exciting new research by Castro et al. (2014). They describe how Cu modulates zinc (Zn) homeostasis (another key metal involved in neurotransmission) in hippocampal neurons, and report that Cu has significant effects on expression of key synaptic proteins, synapsin, and dynamin. Zn and its contribution to neurodegeneration is also the topic covered by Szewczyk (2013), who describes how uncontrolled influx of Zn during traumatic brain injury and stroke, can exacerbate neuronal cell death. In contrast, Zn deficiency may also have a key role in neurodegeneration. This is covered by Szewczyk, with an insight into how changes to Zn transporters and metallothionein may contribute to altered Zn homeostasis in neurodegeneration.

As with Fe, Cu and Zn, less common biometals such as manganese (Mn) appear to have a growing role in neurodegenerative processes. Rather than intoxication from high doses, metals such as Mn can have extraordinary outcomes on complex cortical structures and associated cognitive function even at very low doses. This is covered in depth by Guilarte (2013). These effects may have an important role in neurodegenerative changes in AD and Parkinsonism.

Altered biometal homeostasis is not only a factor in the leading forms or neurodegeneration. They have a key role in many rarer forms of neurodegeneration including childhood neurodegenerative disorders. Parker et al. (2013), report on the role of metals in neuronal ceroid lipofuscinosis, neurodegeneration with brain iron accumulation (NBIA), and additional diseases, further extending the links between genetic mutations and metal abnormalities in these disorders.

Obviously, while this is a rapidly expanding field of research, as illustrated here, we still have much further to go to achieve a major understanding of where these biometal changes fit into the neurodegenerative disease process. Are they prime drivers of disease, significant contributors, a downstream outcome, or (likely) a mix of these? These questions will only be answered through the application of highly sensitive analytical and genetic approaches. Lothian et al. (2013), describe how this can be achieved with the relatively new and rapidly advancing field of metalloproteomics to dissect metal-protein interactions. Chen et al. (2013) then concludes with an excellent description of the powerful genetic model of *C. elegans* and how this is used to pin-point assessment of altered metal homeostasis and its associated genes in a simple but elegant model system.

It has been extraordinarily difficult to get the mainstream fields of neuroscience and medicine to understand the key role biometals have in neurodegeneration. We hope that this Research Topic will help to inform and expand the knowledge on how biometals contribute to neurodegeneration, and inspire others to enter this rapidly growing and exciting field of research. New insights are needed as a basis for innovative therapeutic approaches that hopefully will help to “restore the balance” in these widespread diseases.

## Conflict of interest statement

The authors declare that the research was conducted in the absence of any commercial or financial relationships that could be construed as a potential conflict of interest.
